# Pulmonary fibrosis induced by H5N1 viral infection in mice

**DOI:** 10.1186/1465-9921-10-107

**Published:** 2009-11-12

**Authors:** Jian Qiao, Miaojie Zhang, Jianmin Bi, Xun Wang, Guangcun Deng, Guimei He, Zhihua Luan, Nana Lv, Tong Xu, Lihong Zhao

**Affiliations:** 1 Department of Pathophysiology, College of Veterinary Medicine, China Agricultural University, Beijing 100193, PR China

## Abstract

**Background:**

Inflammatory process results in lung injury that may lead to pulmonary fibrosis (PF). Here, we described PF in mice infected with H5N1 virus.

**Methods:**

Eight-week-old BALB/c mice were inoculated intranasally with 1 × 10^1 ^MID_50 _of A/Chicken/Hebei/108/2002(H5N1) viruses. Lung injury/fibrosis was evaluated by observation of hydroxyproline concentrations, lung indexes, and histopathology on days 7, 14, and 30 postinoculation.

**Results:**

H5N1-inoculated mice presented two stages of pulmonary disease over a 30-d period after infection. At acute stage, infected-mice showed typical diffuse pneumonia with inflammatory cellular infiltration, alveolar and interstitial edema and hemorrhage on day 7 postinoculation. At restoration stage, most infected-mice developed PF of different severities on day 30 postinoculation, and 18% of the survived mice underwent severe interstitial and intra-alveolar fibrosis with thickened alveolar walls, collapsed alveoli and large fibrotic areas. The dramatically elevated hydroxyproline levels in H5N1-infected mice showed deposition of collagen in lungs, and confirmed fibrosis of lungs. The dry lung-to-body weight ratio was significantly increased in infected group, which might be associated with the formation of PF in H5N1-infected mice.

**Conclusion:**

Our findings show that H5N1-infected mice develop the typical PF during restoration period, which will contribute to the investigation of fibrogenesis and potential therapeutic intervention in human H5N1 disease.

## Introduction

Human infections with avian H5N1 influenza virus from birds have occurred successively since the first case was reported in Hong Kong, 1997, which demonstrated that an avian influenza virus could cross the species barrier to infect humans and it is possible that avian influenza virus mutates to be able to transmit among humans [1-3]. As of 12 December 2008, the World Health Organization (WHO) reported that H5N1 virus had caused 390 human infections with 246 people deaths, representing a mortality rate over 60% [4]. WHO has warned of a substantial risk of pandemic of avian influenza in the near future.

Human H5N1 infection is characterized by a severe influenza syndrome, including fever, cough, shortness of breath, and radiological evidence of pneumonia [5,6]. Almost all patients have clinically apparent pneumonia, and death ensues within 9 or 10 days on average after the onset of illness. Most patients die of progressive respiratory failure that is believed to be associated with acute respiratory distress syndrome (ARDS) [1,7]. ARDS is a common, devastating clinical syndrome of acute lung injury with high mortality ranging from 40% to 60% [8,9]. Pathological findings show that 64% of ARDS patients may have pulmonary fibrosis (PF) during convalescence [10]. Owing to the damage of architecture and loss of functional capillary units of the lung, Idiopathic pulmonary fibrosis (IPF) in human can lead to respiratory failure within a few years following diagnostic confirmation, and may correlate with an increased risk of death [11]. PF results as a consequence of many types of severe lung injury and is almost always associated with an inflammatory reaction [12,13]. Some virus infections, such as severe acute respiratory syndrome (SARS) caused by a novel coronavirus, can induce the typical ARDS and PF, and most patients eventually die [14,15]. Also, it is possible that patients infected with H5N1 viruses may suffer from PF as a result of ARDS.

Moreover, if the pandemic outbreak of avian H5N1 influenza in human occurs, as a major consequence of ARDS induced by H5N1 virus, PF may prove to be a thorny sequela for treatment. Because clinical experience with avian H5N1 disease in humans is limited and patients mostly die during early period of pneumonia, we know little about pathological changes in the lung during convalescence. Laboratory animal can provide considerable insight into the pathogenesis of the complex process of fibrosis in the lung. Our previous study showed that H5N1 virus could induce the typical ARDS in mice, which was characterized by about 80% mortality, progressive hypoxemia, and pulmonary inflammatory cellular infiltration, alveolar and interstitial edema and hemorrhage [16]. In this study, we used a mouse model to investigate the pathological changes at restoration stage induced by H5N1 viral infection, to provide basis for future investigation into the pathogenesis of PF in human H5N1 influenza disease.

## Methods

### Virus

The virus used was isolated from chicken in the Hebei Province of China in January 2002, and identified as avian influenza A H5N1 virus by means of hemagglutination inhibition (HI) and neuraminidase inhibition tests. The isolate was designated as A/Chicken/Hebei/108/2002 (H5N1) (Chicken/HB/108). The complete genome sequences (DQ343152, DQ349116, DQ351860, DQ351861, DQ351866, DQ351867, DQ351872, and DQ351873) of the virus can be obtained from GenBank. The virus caused 100% (8 of 8) mortality of 4-wk-old specific pathogen-free (SPF) chicken within 2 days after intravenous infection with 0.2 ml of infectious allantoic fluid at 1:10 dilution. This virus belongs to a highly pathogenic avian influenza virus, according to the criteria of viral virulence [17]. Our previous studies showed that this virus was highly lethal to mice and could cause typical ARDS in mice [16]. The virus was propagated in the allantoic cavities of 10-day-old embryonated SPF chicken eggs at 37°C for 32 h, and third-passage virus was gradient purified and stored at -80°C until use. All manipulations of live viruses were conducted in biosafety level 3+ (BSL-3+) facilities.

### Animals and inoculation with virus

Eight-wk-old female SPF BALB/c mice with body weight of 17-18 g were purchased from Beijing Laboratory Animal Research Center (Beijing, People's Republic of China), and housed in microisolator cages ventilated under negative pressure with HEPA-filtered air. During the experiments, mice were given food and water ad lib.

Our previous study showed that the dose of 1 × 10^1 ^mouse infectious doses (MID_50_) of Chicken/HB/108 H5N1 virus was optimal for the observation of PF. At this dosage, the course of the H5N1 disease was slightly prolonged, and infected mice presented obvious lung injury and PF over a 30-d time period. When animals were infected with a higher dose of 1 × 10^2 ^MID_50_, more than 80% mice died, but with a lower dose of 1 × 10^0 ^MID_50_, the lung injury and PF were not demonstrated. Therefore, in this experiment, mice were inoculated intranasally (50 μl) with 1 × 10^1 ^MID_50 _of Chicken/HB/108 H5N1 virus diluted in sterile saline after lightly anesthetized with diethyl ether. Mice in control group were inoculated with an equivalent dilution (50 μl) of noninfectious allantoic fluid. All manipulations were performed under BSL-3+ laboratory conditions. Animal experiments were conducted according to the established guidelines and approved by the Animal Care Committee of China Agricultural University (Beijing, People's Republic of China).

### Experimental protocols

Two types of experiments were carried out in this study. The first experiment was to investigate the mortality, clinical signs, and the presence and severity of PF in H5N1-infected mice over a 30-d time period. In this experiment, 70 mice were divided randomly into two groups. H5N1-infected group of 50 mice was inoculated with H5N1 virus, and control group of 20 mice received the noninfectious allantoic fluid, as described above. The animals' general behavior and clinical signs, including the food intake, body weight, inactivity, anal temperature (measured with an infrared thermometer) and mortality were monitored daily in each group for 30 days. To observe the food intake, the mice were housed individually, on grids in boxes which were changed twice/week. They were weighed daily and their food intake was measured by offering daily known weights of food and separating and weighing any leftover food in the box at each change. On day 30 after infection, all the mice survived were sacrificed and the whole lungs were removed to assess the degree of PF.

In the second experiment, we characterized the development of lung injury and PF of mice after H5N1 viral infection. Mice were divided randomly into two groups with 40 mice each, as described in experiment 1. Since about 60% mice died between day 6 and day 8 postinoculation (p.i.), larger groups (40 per group) of mice were used. Virus inoculation was the same as this used in experiment 1. Four mice of each group were weighed and euthanized on days 3, 7, 14, and 30 p.i. The whole lungs were removed. Left lobes of lungs were fixed in buffered 10% formalin and embedded in paraffin for histopathological evaluation. The upper parts of right lung lobes were used to determine the lung wet-to-dry weight ratio and dry lung-to-body weight ratio. The remaining lobes of the right lung were stored at -80°C until for determining the lung hydroxyproline contents.

### Lung index measurement

The upper parts of right lungs were excised and weighed before and after oven desiccation at 80°C for 8 hours to calculate indexes according to the following formulas: lung wet-to-dry weight ratio = weight of the whole wet lung/weight of the whole dry lung; the dry lung-to-body weight ratio (%) = weight of the whole dry lung/body weight × 100%.

### Histopathological evaluation

After being fixed in 10% formalin for 7 days, the lung tissues were embedded in paraffin. Serial sections (5 μm) were obtained from the blocks, and three sagittal sections from each lung, i.e., six sections per animal, were stained with hematoxylin and eosin (H-E) and Masson's trichrome. The severity of PF was assessed. All of the specimens were numbered randomly and interpreted by an experimenter blinded to the experimental conditions. Visual grading of PF was performed by determining the Ashcroft's score, with some modification [18,19]. Briefly, the entire fields of each lung section were scanned under an Olympus microscope (Olympus Optical Co., Ltd.) at a magnification of × 100, and each field was visually graded from 0 to 8. Criteria for grading lung fibrosis were as follows: Grade 0 = normal lung; Grade 1 = minimal fibrous thickening of alveolar or bronchiolar walls; Grade 3 = moderate thickening of walls without obvious damage to lung architecture; Grade 5 = increased fibrosis with definite damage to lung structure and formation of fibrous bands or small fibrous masses; Grade 7 = severe distortion of structure and large fibrous areas; Grade 8 = total fibrous obliteration of lung fields. After examination of the whole sections, the mean score of all the fields was taken as the fibrosis score for each animal. The lung fibrosis severity in the mice survived on day 30 p.i. was further classified into 4 grades (from no obvious fibrosis to severe fibrosis) according to fibrosis score ranges as shown in Table 1.

**Table 1:** The severity of lung fibrosis in H5N1-infected mice survived on day 30 after infection

	No obvious fibrosis (0 ≤ MS^**§ **^≤ 1)	Mild fibrosis (1 < MS^**§ **^≤ 3)	Moderate fibrosis (3 < MS^**§ **^≤ 6)	Severe fibrosis (6 < MS^**§ **^≤ 8)
Percentage	27.3% (6 of 22)	22.7% (5 of 22)	31.8% (7 of 22)	18.2% (4 of 22)

The severity of the pulmonary fibrosis was assessed according to the modified Ashcroft's score as described in methods. ^§ ^MS means the mean score of lung fibrosis for each animal (three sagittal sections from each lung, i.e., six sections per animal).

### Lung hydroxyproline measurement

Since collagen deposition is a hallmark of fibrosis and collagen contains significant amounts of hydroxyproline, lung hydroxyproline measurement was used to quantify the severity of fibrosis in this study [20]. According to the manufacturer's instructions, hydroxyproline was measured using the test kit (from Nanjing Jiancheng Bio, China) to estimate the collagen content of the lung. Briefly, lung tissues samples from mice were weighed and hydrolyzed to release hydroxyproline from collagen. Oxidation of the hydroxyproline with chloramines T and the hydroxyproline chromogen was reacted with paradimethylaminobenzaldehyde to develop a pink color. Absorbance of colored products was measured at 550 nm spectrophotometrically and the hydroxyproline contents of samples were calculated by comparing with the standards. Data are expressed as micrograms of hydroxyproline per gram of wet lung weight (μg/g).

### Virus titration

Mice were inoculated intranasally with 1 × 10^1 ^MID_50 _of Chicken/HB/108 viruses (50 μl). Lung tissues were collected on days 1, 3, 5, 6, 8, and 14 p.i. Virus was titrated in embryonated eggs. Mean viral titers were calculated based on three mice per group and expressed as log_10 _EID_50 _per milliliter ± SD.

### Statistical analysis

Data were analyzed with the Statistical Package for Social Science (SPSS, Version 13.0) for Windows and results were expressed as means ± SD. A two-tailed Student t test was used to determine the differences between groups. Data were considered to be statistically significant when p < 0.05.

## Results

### Clinical and gross pathological observation

H5N1-infected mice presented two stages of clinical signs over a 30-d time period after viral inoculation. At the acute stage, the onset and evolution of clinical signs were as follows: on day 3 p.i., slight altered gait, inactivity, ruffled fur, inappetence and weight loss; at days 6 to 8 p.i., more severe inappetence, emaciation, and the visual signs of labored respirations and respiratory distress. Fifty-six percent (28 of 50) of H5N1-infected mice died during this stage, with death peak between days 7 and 8 p.i. The onset of inappetence and inactivity was correlated with loss of body weight, which continued to decline until death. The body temperature slightly declined during the H5N1 infection, and dramatically declined before the mice died. At the restoration stage, the clinical signs in most survived animals resolved gradually on days 9 to 14 p.i., and disappeared almost completely at days 15 to 30. However, 18% (4 of 22) of H5N1-infected mice survived the acute stage but developed late stage disease acceleration as assessed by the presence of inactivity, weight loss, inappetence and emaciation. Macroscopical and histopathological evaluations showed that these mice presented severe PF.

**Figure 1:**
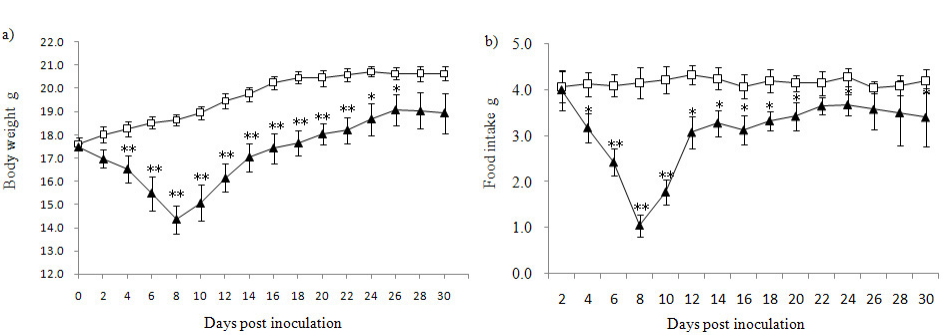
**Body weight (a) and food intake (b) after H5N1 infection in mice**. Uninfected control group (Open squares), and H5N1 virus-infected group (Solid triangles). * p < 0.05 and ** p < 0.01 for the H5N1-infected group vs. control group. Data are presented as means ± SD from 4 mice each group.

Figure 1 shows the changes of body weight and food intake after H5N1 viral infection. The H5N1 infection in mice induced an acute reduction in body weight on days 2 to 8 p.i., and caused a greater decrease in body weight gain on days 10 to 30 p.i. The standard deviations for body weights in infected mice were much greater than those in control mice during the restoration period (on days 10 to 30), indicating the individual differences in the same group. The similar change was also observed in food intake. These data suggested that the survived mice compromised the quality of life over a 30-d time period after viral infection.

**Figure 2:**
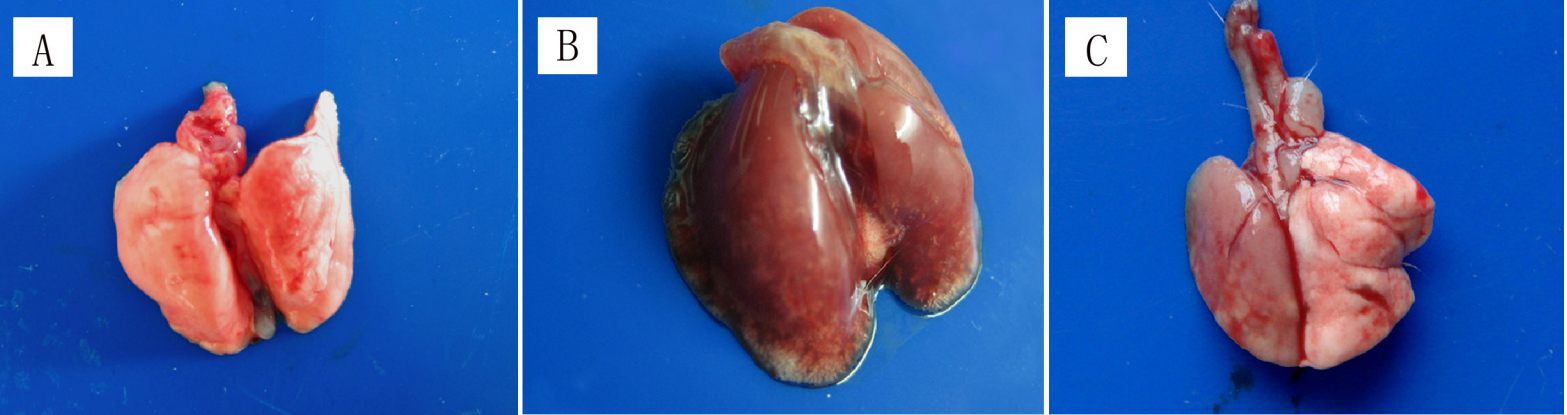
**Macroscopical pathology of lungs after H5N1 infection in mice**. (A) Uninfected control lung. (B) H5N1 virus-infected lung on day 7 p.i., showing the severe edema and hemorrhage. (C) H5N1 virus-infected lung on day 30 p.i., showing fibrosis one side and emphysema on the other side.

The H5N1 virus-infected lungs were significantly heavier than normal lungs (Figure 2A), and showed severe consolidation, edema and hepatization with varying degree of hemorrhage (Figure 2B) on day 7 p.i. On day 30 p.i., some infected animals developed the massive PF with "flesh" appearance in one lung but the severe emphysema on the other (Figure 2C), suggesting that the unilateral lung dysfunction caused by fibrosis was managed to be compensated by the other lung.

### Histopathological findings

**Figure 3:**
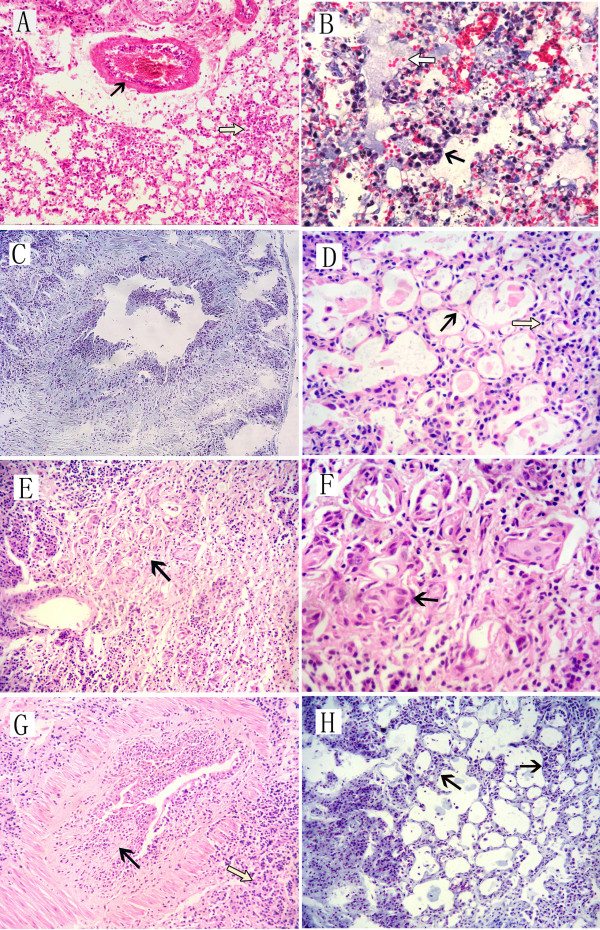
**The lung histopathology as shown by H-E and Masson's trichrome staining after H5N1 infection in mice**. On day 7 p.i., infected mice presented typical diffuse pneumonia with interstitial edema around small blood vessels (Figure 3A, solid arrow) and inflammatory cells in thickened alveolar walls (Figure 3A, open arrow). On day 14 p.i., Masson's staining showed widened alveolar spaces with collagen fibers (Figure 3B, open arrow), and thickened alveolar walls with infiltration of inflammatory cells and hyperplasia of pneumocytes (Figure 3B, solid arrow). On day 30 p.i., severe distortion of structure and diffuse fibrous areas were observed in lung fields (Figure 3C) at low magnification. Some animals displayed typical interstitial fibrosis in lungs, characterized by greatly thickened alveolar walls with cell proliferation and matrix accumulation in interstitial space (Figure 3D, solid arrow). The diffuse intra-alveolar fibrosis was a common finding, with an excessive collagen deposition and cell proliferation in airspaces that obliterated the alveolar spaces and severely distorted the structure (Figure 3E and 3F, solid arrows; Figure 3D and 3G, open arrows). The thickened bronchiolar walls with proliferated fibroblasts lead to bronchiolar stenosis (Figure 3G, solid arrow). In addition, focal alveolar collapse (Figure 3H, solid arrows) and alveolar ectasia (Figure 3D and 3H) were observed simultaneously in some sections. Original magnification: A, E, G, H-E × 200; D, F, H-E × 400; C, Masson × 100; H, Masson × 200; B, Masson × 400.

Histopathological lesions could be subclassified into two consecutive phases, an initial acute exudative phase and a final fibrotic phase, although considerable overlap existed in histological findings between two phases. The histopathological changes of lung injury and fibrosis with H-E and Masson's stain are shown in Figure 3.

At the initial exudative stage, the infected mice presented typical diffuse pneumonia and the lesions associated with ARDS on day 7 p.i. There were interstitial edema around the small blood vessels and adherence of inflammatory cells to wall of the small vessel (Figure 3A). Alveolar walls were thickened, and inflammatory cells infiltrated in the interstitial and intra-alveolar spaces (Figure 3A). On day 14 p.i., Masson's stain showed that alveolar spaces were widened and filled with collagen fibers (Figure 3B), indicating that proliferative fibroblastic lesions may develop in future. Alveolar walls were generally thickened with infiltration of inflammatory cells and hyperplasia of type II pneumocytes. Hemorrhage was also observed in interstitium and intra-alveolar space (Figure 3B).

At the final fibrotic stage, the survived mice developed PF of different severities on day 30 p.i. According to Ashcroft's method [18,19], severity scores for lung fibrosis in the survived mice are given in Table 1, which showed that 23% (5 of 22) mice had minimal fibrosis, 32% (7 of 22) had moderate fibrosis, and 18% (4 of 22) underwent severe fibrosis. At low power magnification, severe distortion of structure and diffuse fibrous areas were observed in lung fields (Figure 3C). The severely affected animals had the typical interstitial fibrosis in lungs, exhibiting greatly thickened alveolar walls with proliferation of cells and the accumulation of matrix in interstitial space bounded by epithelial and endothelial basement membranes (Figure 3D). The diffuse intra-alveolar (airspace) fibrosis was a common finding at this stage. Large fibrous areas were seen with an excessive collagen deposition and cell proliferation in airspaces, which obliterated the alveolar spaces, organized lung parenchyma and severely distorted lung (Figure 3D, 3E, 3F and 3G). Bronchiolar wall was thickened, and numerous fibroblasts proliferated and protruded into the lumen, leading to bronchiolar stenosis (Figure 3G, solid arrow). In addition, the focal alveolar collapse secondary to apposition of the alveolar walls was observed in some section (Figure 3H), and both alveolar ectasia and airspace fibrosis were also found in the same field of lung (Figure 3H).

Lung tissues from control group showed a normal architecture with opened patterns of alveolar spaces, thin-lined alveolar septa, only a few alveolar macrophages and minimal collagen deposition.

### Hydroxyproline content

**Figure 4:**
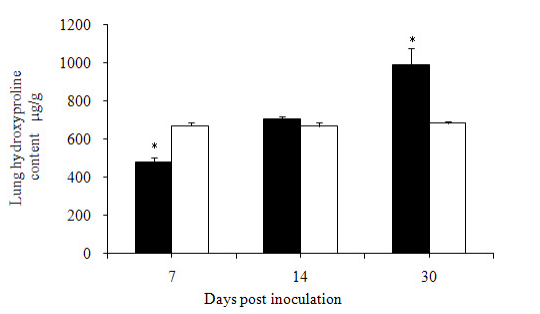
**Hydroxyproline contents of lungs after H5N1 infection in mice**. The levels of lung hydroxyproline in H5N1 virus-infected mice (Solid bars) and control mice (Open bars) were determined using test kit, and shown as means ± SD from 4 mice each group. * p < 0.05 compared with those in control mice.

Hydroxyproline, the amino acid that is found almost exclusively in collagen, can reflect the collagen deposition in lungs [20]. Therefore, lung hydroxyproline measurement can provide a direct measurement of the formation of PF. In this study, lung hydroxyproline contents, as shown in Figure 4, were measured at days 7, 14, and 30 p.i. On day 7 p.i., H5N1 group showed a significant decrease of the lung hydroxyproline contents compared with control animals, which might reflect the development of severe lung edema in infected mice. The lung hydroxyproline levels returned to normal on day 14 p.i., and increased by almost two-fold on day 30 p.i. compared with that in control mice. The change of hydroxyproline contents was consistent with our histopathological findings which demonstrated the formation of PF on day 30 after infection.

### Lung wet-to-dry weight ratio and dry lung-to-body weight ratio

**Figure 5:**
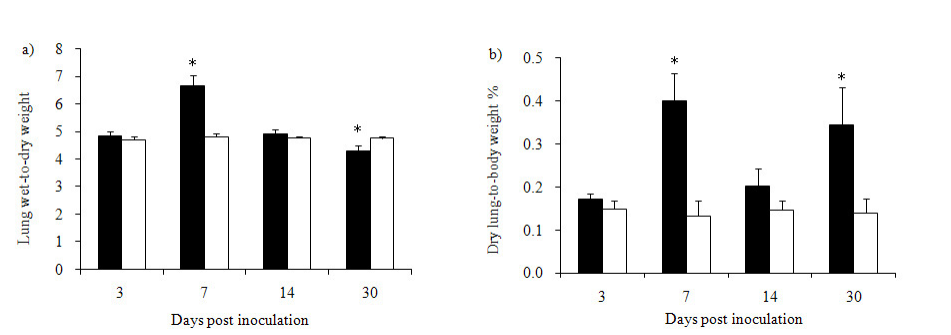
**Lung wet-to-dry weight ratios (a) and dry lung-to-body weight ratios (b) after H5N1 infection in mice**. Uninfected control group (Open bars), and H5N1 virus-infected group (Solid bars). * p < 0.05 for the H5N1 virus-infected group vs. control group. Values are presented as means ± SD from 4 mice each group.

Figure 5 shows the effect of H5N1 viral infection on lung wet-to-dry weight ratios and dry lung-to-body weight ratios. In H5N1-infected mice, two ratios did not change obviously on day 3 p.i., but dramatically elevated on day 7 p.i., suggesting the severe edema and inflammatory exudates of the lung. Both ratios of infected mice returned to control levels on day 14 p.i., indicating that edema and inflammatory exudates had been reabsorbed. On day 30 p.i., the survived mice showed the significantly decreased lung wet-to-dry weight ratios but dramatically increased dry lung-to-body weight ratios, which might be associated with the formation of PF in H5N1-infected mice.

### Replication of H5N1 viruses in mouse lungs

**Figure 6:**
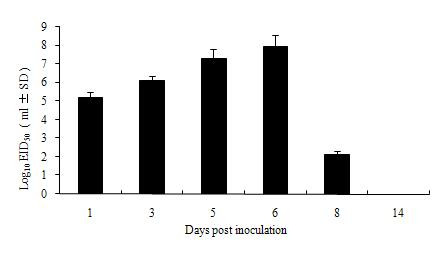
**Replication of H5N1 virus in mouse lungs**. Mice were inoculated intranasally with 1 × 10^1 ^MID_50 _of Chicken/HB/108 virus. Viruses were titrated in embryonated eggs. Mean viral titers (Solid bars) were calculated based on three mice per group.

H5N1 viral infection made high titers of viruses in the lungs on days 5 and 6 p.i., as shown in Figure 6. Peak viral titer appeared on day 6 p.i., and viruses were below the detectable level on day 14 p.i.

## Discussion

In this report, we focused the development of PF in mice after intranasal infection with 1 × 10^1 ^MID_50 _of Chicken/HB/108 H5N1 virus.

The typical ARDS was observed in the early period after H5N1 viral infection. Most of the infected mice exhibited clinical signs of respiratory distress with 56% of the mice died on days 6 to 8 p.i. Macroscopical observation showed that the infected mice had highly edematous lungs on day 7 p.i., which were also demonstrated by the dramatically increased lung wet-to-dry weight ratios. At the microscopical level, infected mice exhibited typical diffuse pneumonia and obvious ARDS-associated pathological changes, including fully developed bronchiolitis, and peribronchiolar pneumonia characterized by inflammatory cellular infiltration, interstitial and alveolar edema, and hemorrhage. These data are consistent with our previous studies [16].

Most infected mice developed PF gradually at late stage. On day 30 p.i., 18% of the survived mice underwent severe fibrosis according to Ashcroft's method [18,19]. Typical interstitial and intra-alveolar fibrosis was observed, with thickened alveolar walls, collapsed alveoli and large organized areas. Both Intra-alveolar fibrosis and ectasia were showed simultaneously in the same lung specimens. Reactive hyperplasia of pneumocytes was present in organizing phase. Bronchiolar stenosis, resulting from the thickened bronchiolar walls with proliferated fibroblasts, was also found under light microscope. Moreover, as an important index of fibrosis, the hydroxyproline levels increased significantly in H5N1-infected mice as compared to control group on day 30 p.i., which showed the deposition of collagen in lungs, and confirmed the fibrosis of lungs.

During the restoration period, the clinical signs in most animals resolved gradually on days 9 to 14 p.i., and disappeared almost completely on days 15 to 30. However, 18% (4 of 22) of H5N1-infected mice survived the acute stage but develop the accelerated disease progression at late stage, as assessed by the presence of inactivity, weight loss, inappetence and emaciation. Macroscopical and histopathological evaluations showed that these mice presented severe PF. These data show that the survived H5N1-infected mice with fibrotic lung severely compromised the quality of life. It is well established that the lung can be subjected to inflammatory reactions and return to its pre-inflammatory state [12,13]. If viral pneumonia, including H5N1 viral infection, causes a more intense inflammatory process in the lung, the fibrosis, rather than resolution, might become an inevitable outcome. During experiments, we found no secondary bacterial infection, and titers of viruses in the lungs were below the detectable levels on day 14 p.i. Our data is likely to reflect the clinical and pathological evolution from ARDS to fibrosis in lungs subsequently to H5N1 infection in mice.

For human, fibrosis of the lung is correlated with respiratory failure and an increased risk of death [11,21]. Several postmortem reports on patients with H5NI influenza have demonstrated the presence of organizing diffuse alveolar damage with interstitial fibrosis [22]. So, under the pressure of pandemic human-avian influenza in the near future, we must be prepared to prevent the spread of this virus and find effective method to reverse the relentless progress of PF induced by H5N1 viral infection. Our research may provide a valuable mouse model to study the development of this disease and to search for treatment of the human H5N1 influenza.

At present, the pathogenesis of PF is not fully understood. Some studies showed that the cytokines, such as transforming growth factor-β (TGF-β), interleukin-10 (IL-10), and platelet-derived growth factor (PDGF), appeared to have an important role in the control of fibroblast activity in vitro, and have been implicated in the pathogenesis of PF [23,24]. PF is difficult to treat clinically and few drugs can be useful. Corticosteroid therapy like dexamethasone is the current first-line choice to prevent this disease because of its anti-inflammatory effects [25]. Laboratory studies showed that corticosteroids might prevent the collagen overexpression and decrease the up-regulated cytokines including TGF-β in bleomycin-induced PF model [26,27]. Our further study will use the mouse model to focus on the role of cytokines in pathogenesis of PF and the effect of some drugs on PF induced by H5N1 virus.

## Conclusion

Our findings show that H5N1-infected mice develop typical PF during restoration period as a result of ARDS, which will contribute to the investigation of fibrogenesis and potential therapeutic intervention in human H5N1 disease.

## List of abbreviations

ARDS: acute respiratory distress syndrome; PF: pulmonary fibrosis; IPF: idiopathic pulmonary fibrosis; SARS: severe acute respiratory syndrome; Chicken/HB/108: A/Chicken/Hebei/108/2002 (H5N1); SPF: specific pathogen-free; MID_50_: mouse infectious doses; p.i.: postinoculation; H-E: hematoxylin and eosin; TGF-β: transforming growth factor-β; IL-10: interleukin-10; PDGF: platelet-derived growth factor.

## Competing interests

The authors declare that they have no competing interests.

## Authors' contributions

JQ and LZ conceived the study, conducted H5N1 viral infection and finalized the manuscript. MZ and JB carried out the histopathological studies and drafted the manuscript. XW, GD and GH carried out the animal studies. ZL, NL and TX participated in completing the study. All authors read and approved the final manuscript.

## References

[B1] Beigel JH Farrar J Han AM Hayden FG Hyer R de Jong MD Lochindarat S Nguyen TK Nguyen TH Tran TH Nicoll A Touch S Yuen KY Avian influenza A (H5N1) infection in humans N Engl J Med 2005 353 1374 1385 10.1056/NEJMra052211 16192482

[B2] Iwami S Takeuchi Y Liu X Avian-human influenza epidemic model Math Biosci 2007 207 1 25 10.1016/j.mbs.2006.08.001 17010999

[B3] Claas EC Osterhaus AD van Beek R De Jong JC Rimmelzwaan GF Senne DA Krauss S Shortridge KF Webster RG Human influenza A H5N1 virus related to a highly pathogenic avian influenza virus Lancet 1998 351 472 477 10.1016/S0140-6736(97)11212-0 9482438

[B4] World Health Organization. Cumulative number of confirmed human cases of avian influenza A/(H5N1) reported to WHO http://www.who.int/csr/disease/avian_influenza/country/cases_table_2008_12_16/en/index.html Date last updated: December 16 2008. Date last accessed: December 16 2008

[B5] Yu H Shu Y Hu S Zhang H Gao Z Chen H Dong J Xu C Zhang Y Xiang N Wang M Guo Y Cox N Lim W Li D Wang Y Yang W The first confirmed human case of avian influenza A (H5N1) in Mainland China Lancet 2006 367 84 10.1016/S0140-6736(05)67894-4 16399159

[B6] Bay A Etlik O Oner AF Unal O Arslan H Bora A Davran R Yuca SA Dogan M Radiological and clinical course of pneumonia in patients with avian influenza H5N1 Eur J Radiol 2007 61 245 250 10.1016/j.ejrad.2006.10.006 17110072

[B7] Schunemann HJ Hill SR Kakad M Bellamy R Uyeki TM Hayden FG Yazdanpanah Y Beigel J Chotpitayasunondh T Del Mar C Farrar J Tran TH Ozbay B Sugaya N Fukuda K Shindo N Stockman L Vist GE Croisier A Nagjdaliyev A Roth C Thomson G Zucker H Oxman AD WHO Rapid Advice Guidelines for pharmacological management of sporadic human infection with avian influenza A (H5N1) virus Lancet Infect Dis 2007 7 21 31 10.1016/S1473-3099(06)70684-3 17182341 PMC7106493

[B8] Dulu A Pastores SM Park B Riedel E Rusch V Halpern NA Prevalence and mortality of acute lung injury and ARDS after lung resection Chest 2006 130 73 78 10.1378/chest.130.1.73 16840385

[B9] Luecke T Muench E Roth H Friess U Paul T Kleinhuber K Quintel M Predictors of mortality in ARDS patients referred to a tertiary care centre: a pilot study Eur J Anaesthesiol 2006 23 403 410 10.1017/S0265021505001870 16469204

[B10] Martin C Papazian L Payan MJ Saux P Gouin F Pulmonary fibrosis correlates with outcome in adult respiratory distress syndrome. A study in mechanically ventilated patients Chest 1995 107 196 200 10.1378/chest.107.1.196 7813276

[B11] Molina-Molina M Badia JR Marin-Arguedas A Xaubet A Santos MJ Nicolas JM Ferrer M Torres A Outcomes and clinical characteristics of patients with pulmonary fibrosis and respiratory failure admitted to an intensive care unit. A study of 20 cases Med Clin (Barc) 2003 121 63 67 10.1157/13048922 12828887

[B12] Ward PA Hunninghake GWL Lung inflammation and fibrosis Am J Respir Crit Care Med 1998 157 S123 129 9563771 10.1164/ajrccm.157.4.nhlbi-10

[B13] Reynolds HY Lung inflammation and fibrosis: an alveolar macrophage-centered perspective from the 1970s to 1980s Am J Respir Crit Care Med 2005 171 98 102 10.1164/rccm.200406-788PP 15557133

[B14] Cheung OY Chan JW Ng CK Koo CK The spectrum of pathological changes in severe acute respiratory syndrome (SARS) Histopathology 2004 45 119 124 10.1111/j.1365-2559.2004.01926.x 15279629 PMC7194176

[B15] Egan JJ Woodcock AA Stewart JP Viruses and idiopathic pulmonary fibrosis Eur Respir J 1997 10 1433 1437 10.1183/09031936.97.10071433 9230225

[B16] Xu T Qiao J Zhao L Wang G He G Li K Tian Y Gao M Wang J Wang H Dong C Acute respiratory distress syndrome induced by avian influenza A (H5N1) virus in mice Am J Respir Crit Care Med 2006 174 1011 1017 10.1164/rccm.200511-1751OC 16917113

[B17] Swayne DE Halvorson DA Saif IYM, Barnes HJ, Glisson JR, Fadly AM, McDougald LR, Swayne DE Influenza Diseases of Poultry 2003 Iowa State University Press, Ames, Iowa 135 170

[B18] Ashcroft T Simpson JM Timbrell V Simple method of estimating severity of pulmonary fibrosis on a numerical scale J Clin Pathol 1988 41 467 470 3366935 10.1136/jcp.41.4.467 PMC1141479

[B19] Ouchi H Fujita M Ikegame S Ye Q Inoshima I Harada E Kuwano K Nakanishi Y The role of collagenases in experimental pulmonary fibrosis Pulm Pharmacol Ther 2008 21 401 408 10.1016/j.pupt.2007.10.006 18060817

[B20] Woessner JF Jr The determination of hydroxyproline in tissue and protein samples containing small proportions of this imino acid Arch Biochem Biophys 1961 93 440 447 10.1016/0003-9861(61)90291-0 13786180

[B21] Blivet S Philit F Sab JM Langevin B Paret M Guerin C Robert D Outcome of patients with idiopathic pulmonary fibrosis admitted to the ICU for respiratory failure Chest 2001 120 209 212 10.1378/chest.120.1.209 11451840

[B22] To KF Chan PK Chan KF Lee WK Lam WY Wong KF Tang NL Tsang DN Sung RY Buckley TA Tam JS Cheng AF Pathology of fatal human infection associated with avian influenza A H5N1 virus J Med Virol 2001 63 242 246 10.1002/1096-9071(200103)63:3<242::AID-JMV1007>3.0.CO;2-N 11170064

[B23] Allwn JT Spiter MA Growth factors in idiopathic pulmonary fibrosis: relative roles Respir Res 2002 3 13 21 11806848 10.1186/rr162 PMC64811

[B24] Coker RK Laurent GJ Pulmonary fibrosis: cytokines in the balance Eur Respir J 1998 11 1218 1221 10.1183/09031936.98.11061218 9657557

[B25] American Thoracic Society Idiopathic pulmonary fibrosis: diagnosis and treatment. International consensus statement. American Thoracic Society (ATS), and the European Respiratory Society (ERS) Am J Respir Crit Care Med 2000 161 646 664 10673212 10.1164/ajrccm.161.2.ats3-00

[B26] Li HP Li X He GJ Yi XH Kaplan AP The influence of dexamethasone on the proliferation and apoptosis of pulmonary inflammatory cells in bleomycin-induced pulmonary fibrosis in rats Respirology 2004 9 25 32 10.1111/j.1440-1843.2003.00523.x 14982598

[B27] Dik WA McAnulty RJ Versnel MA Naber BA Zimmermann LJ Laurent GJ Mutsaers SE Short course dexamethasone treatment following injury inhibits bleomycin induced fibrosis in rats Thorax 2003 58 765 771 12947134 10.1136/thorax.58.9.765 PMC1746812

